# Complete regression of intrahepatic cholangiocarcinoma after right portal vein ligation. Case report

**DOI:** 10.1016/j.ijscr.2024.109580

**Published:** 2024-03-24

**Authors:** Doru-Florian-Cornel Moga, Gabriela-Ariadna Gavrilă, Andreea-Alina Dan, Cătălin-Gabriel Smarandache

**Affiliations:** a Clinical Department of Surgery, Military Clinical Emergency Hospital Sibiu and Lucian Blaga University Sibiu, Romania; b Medical Analysis Laboratory, Military Clinical Emergency Hospital Sibiu and Lucian Blaga University Sibiu, Romania; c Department of Radiology, Military Clinical Emergency Hospital Sibiu, Romania; d Faculty of Medicine, Carol Davila University of Medicine and Pharmacy Bucharest, Romania

**Keywords:** Cholangiocarcinoma, Tumor regression, Portal vein ligation, Case report

## Abstract

**Introduction:**

Spontaneous tumor regression is an extremely rare phenomenon in the oncology field.

**Presentation of case:**

We present the case of a 72-years-old male patient presenting with a bulky hepatic tumor mass located in segment V and extending into segments IVb and VI with MRI features of atypical cholangiocarcinoma with a liver metastasis in segment III. In first surgical step, excision of the metastasis, and ligation of the right portal vein was done. A new MRI examination performed 5 weeks later shows significant tumor regression, and 2 weeks later, during the second surgery, the tumor was not found. Under these conditions we performed a limited segment V liver resection, in the area indicated by the radiologist as the site of the tumor. No viable malignant cells existed in the tumor specimen, and a third MRI examination didn't identify any residual tumor.

**Discussion:**

From our literature study this is the only case of complete tumor regression of an intrahepatic cholangiocarcinoma following portal vein ligation. We believe the portal vein ligation resulted in a marked regression/deficiency in the tumor blood supply.

**Conclusion:**

Serial MRI examinations demonstrated the regression of intrahepatic cholangiocarcinoma after portal vein ligation. Intrahepatic cholangiocarcinoma should be included in the tumors that could extremely rarely spontaneously regress.

## Highlights

• Spontaneous tumor regression is an extremely rare phenomenon in oncology and thus, we present a case of a right liver lobe tumor with imaging features of atypical cholangiocarcinoma with a single left lobe metastasis that disappeared after right portal vein ligation.
• We decided to approach the case based on a two-stage hepatectomy. The first surgical step was metastasectomy and right portal vein ligation.
• To our surprise, at the 5th week follow-up, the primary tumor significantly reduced, without any adjuvant specific chemotherapy.
• Given these conditions, we performed a limited segment V resection, and neither the histopathology report, nor the follow-up MRI examination identified any residual tumor.

## 1 Introduction

Cholangiocarcinoma (CCA) accounts for approximately 10 % of all hepatobiliary tumors and represents 3 % of all newly-diagnosed malignancies worldwide [1]. However, CCA equally accounts for 13 % of the total cancer mortality worldwide [2]. This rare tumor arises from the biliary epithelium and can develop at any level of the intra- and extrahepatic biliary tree. According to its location, it is classified as intra-hepatic, peri-hilar, or distal, representing three distinct entities in terms of biology, treatment options, and prognosis [1,3]. Intrahepatic cholangiocarcinoma (i-CCA) arises from the biliary epithelium proximal to the second-degree bile ducts [1].

The incidence of i-CCA is increasing worldwide; however, research is hampered by several of the condition's characteristics [4]. Although the etiology of CCA is unclear, it is related to hepatolithiasis, liver-fluke infection, liver cirrhosis, chronic hepatitis B or C, and inflammation of the bile duct, such as by primary biliary cholangitis or primary sclerosing cholangitis [4].

The latest 5th WHO classification further subcategorizes i-CCA into the large duct type and the small duct type based on the origin of the cells (intrahepatic large bile ducts and peribiliary glands for the large duct type, and small bile ducts and bile ductules/hepatic progenitor cells for the small duct type) [5]. Magnetic resonance imaging (MRI) may have a role in subcategorizing i-CCA subtype preoperatively and noninvasively [5].

A proportion of i-CCAs may develop multiple liver lesions (“liver metastases”), with no evidence of extrahepatic disease [6].

Owing to peripheral liver growth of i-CCA and to the normal hepatic function, patients often present with non-specific symptoms such as vague right upper quadrant pain, weight loss, and fatigue, while jaundice is less common compared with perihilar-CCA patients (15–16 % of cases) [1]. The work has been reported in line with the SCARE criteria [7,8].

## 2 Case presentation

We present the case of a 72-year-old male patient, without significant personal pathological history, who, on the background of discrete abdominal painful complaints, undergoes an MRI examination (13 April 2023).

**Fig. 1:**
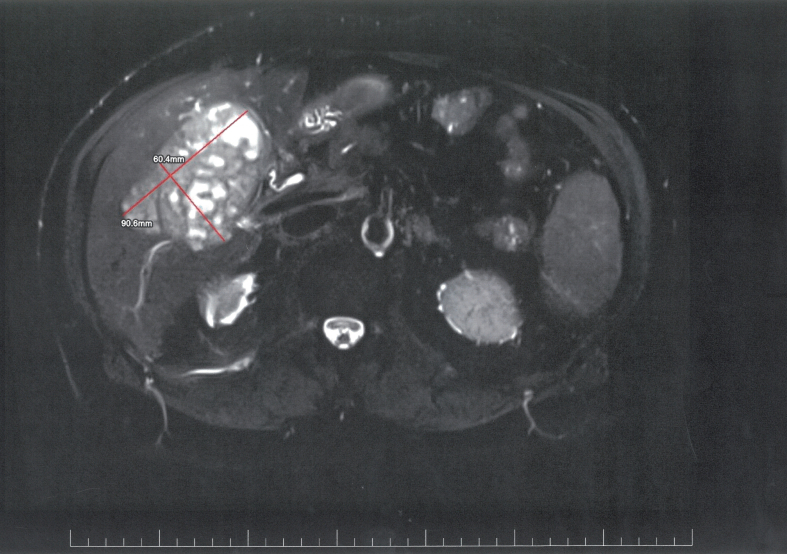
T2-FS axial - large mass involving segment V, VII, VIII (partially included in the presented image), inhomogeneous with multiple cystic and serpiginous fluid spaces and well-defined margins, with no infiltrative pattern and no dilatations of the intrahepatic biliary ducts.

**Fig. 2:**
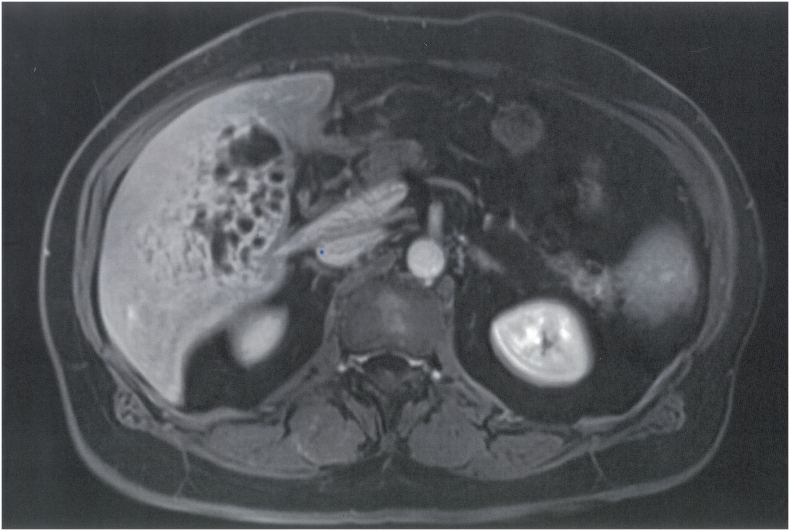
T1-FS axial delayed phase enhancement (5 min) - the mass displays enhancement of the septa delimitating the fluid spaces, having an overall areolar, “sponge-like” structure.

**Fig. 3:**
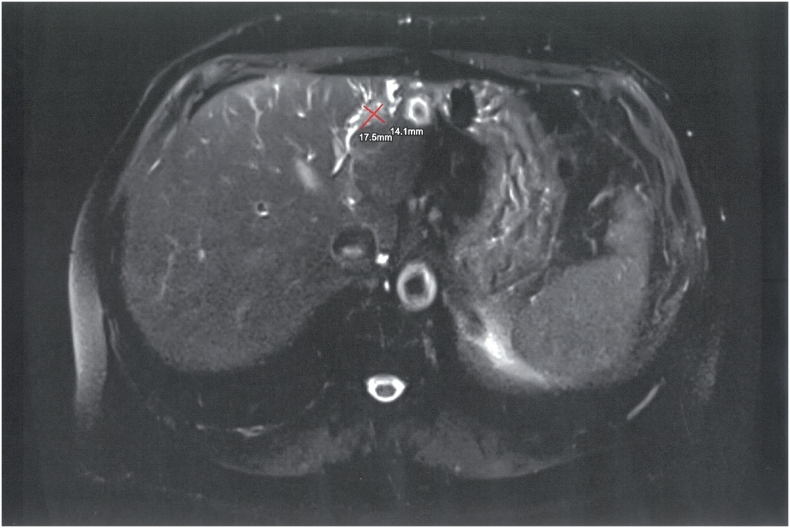
Small nodular homogenous lesion in segment III, mildly hyperintense in T2-FS axial and intense diffusion restriction (not shown), associated with moderate dilatations of the peripheral intrahepatic biliary radicles-suggestive of secondary lesion.

Voluminous tumoral mass measuring 95/56/91 mm (antero-posterior/transverse/cranio-caudal diameter) is noted in segment V, extending into segment IVb and VI with moderate T2 hyperintensity (Fig. 1), T1 hypointensity compared to the liver parenchyma, also seen in In/OPPhase (not shown) with no significant changes between these sequences, highly restricted in DWI/ADC (not shown), including at high B values (B800). The tumor is well defined with a thin wall, best appreciated at the visceral surface and it has an inhomogenous inner structure with multiple round and serpiginous fluid areas with inner denser foci, interspaced with a solid component, generating an areolar pattern. Dynamic iv contrast images (Fig. 2) show gradual uptake of the solid component that correlates with diffusion restriction, while the fluid components display less diffusion restriction and no enhancement. The tumor does not invade the surrounding structures and it is associated with dilatations of peripheral biliary radicles distal to the tumor. Associated with the tumor, a nodular lesion was also seen in segment III, which iso/discreetly hyperintense in T2-FS and isointense in all the other unenhanced images and was mostly apparent due to the consecutive dilatations of the distal small biliary branches. (Fig. 3).

**Fig. 4:**
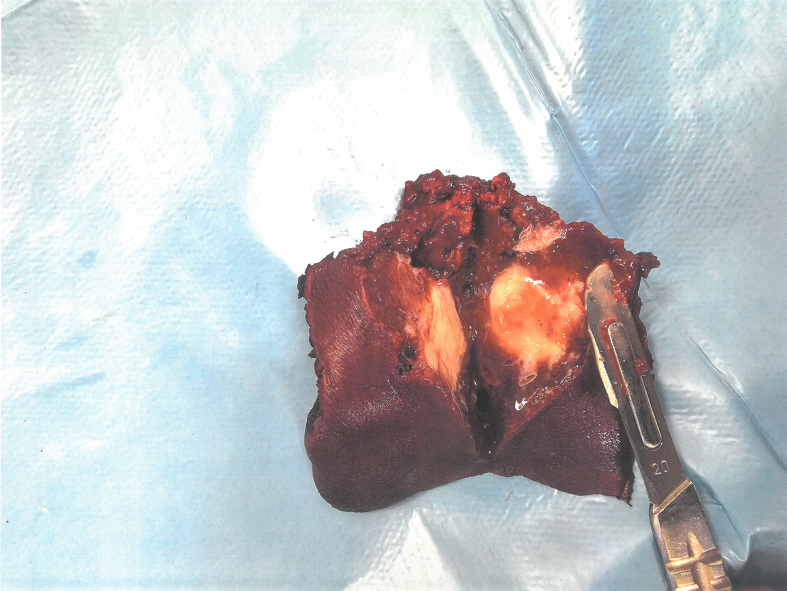
Atypical segment III resection.

On April 25th 2023, the first surgery was performed. Atypical resection of the segment III (Fig. 4) and right portal vein ligation was performed by open approach. The pathological an immunohistochemical staining confirmed that the hepatic tumor resected from segment III was a mucoepidermoid subtype of poorly differentiated (G3) cholangiocarcinoma metastasis.

**Fig. 5:**
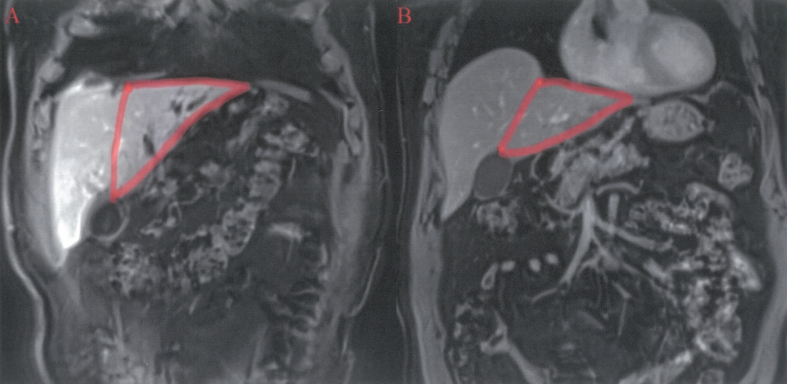
The sFLR pre right portal vein ligation was 17.8 % and post right portal vein ligation was 14.71 %.

The patient undergoes a follow-up MRI examination on May 31st 2023 which, to our surprise, showed a marked involuted tumoral mass, which appeared localized only in segment V. In addition, the expected hypertrophy of the left liver was not obtained (Fig. 5).

Second surgery was performed on June 16th, 2023. Both clinical examination and ultrasound failed to identify with certainty the tumor in segment V. Under these conditions a limited resection was performed of segment V, in the area indicated by the radiologist as being the site of the tumor, together with and hepatic pedicle lymphadenectomy. The anatomopathological results showed only foci of low-grade intraepithelial neoplasia, without malignant lesions, and two lymph nodes without carcinomatous metastases.

**Fig. 6:**
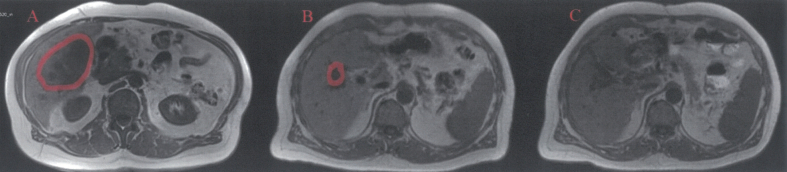
A- T1-ax the tumor at the initial presentation seen as a hypointense mass with areolar “sponge-like” pattern involving the aforementioned segments. B) tumor regression after the right portal vein ligation. C) complete tumor excision after segment V atypical resection, with small fluid postsurgical collection in the tumor bed.

Follow-up MRI examination on June 23rd, 2023 confirmed the absence of hepatic tumor lesions (Fig. 6).

Slowly favorable postoperative evolution, with a small biliary fistula that closed spontaneously. The patient was discharged on the 15th postoperative day.

The patient underwent a follow up MRI in the 15th of November 2023 which did not show any recurrence both at the site of metastasectomy and also in the tumor bed.

## 3 Discussion

### 3.1 Surgical considerations about intrahepatic cholangiocarcinoma

Although the incidence is low, i-CCA represents the second-most common primary liver cancer after hepatocellular carcinoma (HCC) [4].

Having an insidious evolution, the diagnosis is frequently established in advanced stages, even in non-operable stages. At presentation, about 50 % of patients have unresectable disease and another 30 % will be found unresectable during surgical exploration [9].

Surgical resection is the only potentially curative therapy, even if the 5-year overall survival is poor, ranging between 15 % and 45 % owing to several risk factors like multifocal disease, margin status, vascular infiltration, and lymph nodes' involvement [4]. Adjuvant chemotherapy did not improve patient survival significantly [10]. To date, it has been verified that patients with small duct i-CCA tend to exhibit better survival outcome and less recurrence than those with large duct [5].

The objective of surgery is to achieve a radical, margin-negative (R0) tumor resection [1], while preserving adequate liver function [11]. In our case, although we considered upfront that a right extended hepatectomy, including IVb segmentectomy associated with a segment III metastasectomy would be too extensive, we still proceeded in assessing the standardized future liver remnant sFLR before and after portal vein ligation, using the following formulas:

$$\text{sFLR}\%=\mathrm{FLR}/\text{TELV},\text{TELV}=\text{total estimated liver volume calculated with the formula}-794=1267\text{xBSA},\mathrm{BSA}\;\text{body surface area calculated}\;\mathrm{at}\;1,81m^{2}.$$

The postoperative sFLR was 14.71 %, which, in normal liver, is below the accepted threshold of 20 % which is considered the minimum safe volume needed after extended resection.

Central localization of i-CCA is quite common and the need for extensive hepatectomies, complex liver resections are often necessary. For this reason has necessitated the use of adjuvant methods to preserve sufficient liver remnant after resection. A sufficient future liver remnant is critical in decreasing postoperative morbidity and mortality [2,9]. In case of a non-sufficient future liver remnant, are used portal vein ligation or embolization, associating liver partition for staged hepatectomy, vascular resections [1,9].

Although resection traditionally offers the only chance for long-term survival and cure for patients with i-CCA and perihilar cholangiocarcinoma, there is an emerging role of liver transplantation in selected patients with biliary tumors which are otherwise considered unresectable [3]. The advantage of liver transplantation relates to the best chance of achieving a R0 resection but is otherwise compromised by the immunosuppression and the risk of tumor recurrence [3].

Although there is no evidence supporting the therapeutic role of lymphadenectomy in i-CCA patients, current guidelines recommend lymphadenectomy of portal vein in order to achieve a complete staging and for the crucial prognostic role of nodal involvement [1,12]. A significant positive association between the number of lymph node metastasis and poor outcomes was observed [11]. T status, N status, and tumor recurrence seem to be the most important prognostic factors after resection [10].

In the case presented by us we have not performed a biopsy of the primary tumor because we regarded it unnecessary, considering that we would obtain a similar result from the metastasis analysis. In a retrospective view, a biopsy would have provided us with an additional argument that the diagnosis supported by the MRI interpretation was the correct one.

### 3.2 The role of MRI in the diagnosis of i-CCA

Due to its excellent soft tissue contrast, MRI is the imaging modality of choice for diagnosis and staging of cholangiocarcinoma, having the accuracy similar to that of contrast enhanced CT and direct cholangiography [13].

According to the Liver Cancer Study group of Japan, i-CCA can be classified considering the morphological aspect and tumor growth pattern in mass forming type (the most frequent-approximately 80 %), periductal infiltrating type, or intraductal growing type [5].

MRI can have a role in setting the differential diagnosis between CCA and other primary and secondary tumors, especially HCC, and also in discriminating the subtypes, although there is a significant overlap of the imaging aspects, since both large duct and small duct i-CCA subtypes could present as mass forming. Most I-CCA have varying degrees of stromal fibrosis which is a key feature that allows proper diagnosis.

Typically, the mass forming type appears as T2-hyperintense, T1-hypointense tumor, well defined but with irregular borders, with inhomogenous internal structure having a peripheral tissue component which displays intense diffusion restriction, and central areas of necrosis, with no restriction in DWI/ADC. Dynamic contrast enhancement pattern with extracellular agent mirrors the morphological plain features, showing prominent peripheral wash-in, with gradual centripetal enhancement especially on delayed image acquisition (at 10 min).

Atypical forms comprise two types: hypervascular and mucinous i-CCA. The former is usually a small lesion seen in cirrhotic liver, characterized by less fibrosis and more prominent intravascular stroma, which explains the behavior after iv contrast administration: no washout, progressive enhancement and no pseudocapsule. The latter is characterized by rich mucin production organized in large mucinous lakes which harbor grouped cancer cells, generating an intense hyper T2 signal, intralesional septa with progressive enhancement [14].

In our case, the general morphology of the tumor, especially the septa, the multiple areolar foci, and pattern of enhancement, are suggestive of the latter type of atypical I-CCA, which is o very rare occurrence.

### 3.3 Liver tumor regression (TR)

Spontaneous regression (SR) of cancer is the partial or complete disappearance of a malignant tumor in the absence of all treatment, or in the presence of therapy which is considered inadequate to exert a significant influence on the neoplastic disease [15]. When maximal diameter was used as the evaluated variable, partial response was defined as a decrease greater than 50 % and minor response as a decrease greater than 20 % [16].

Historically, most cases of SR have been reported in renal cell carcinoma, neuroblastoma, melanoma [17], choriocarcinoma, bladder carcinoma, leukemia, and some forms of Hodgkin's disease [18]; however, in recent reports, lung and liver cancers are also considered to be the types of cancers which may regress [19].

In the literature reviewed, we found no reports of regression of an i-CCA after portal vein ligation. Moreover, we found only one article published in 1996 reporting SR of an i-CCA [20]. Under these conditions, we analyzed the cases of HCC regression, found in literature, considering that we might find possible explanations for our case. Seemingly, SR of HCC was considered to be an extremely infrequent phenomenon, with an estimated incidence of 1 in 60,000–100,000 cases [21,22].

Of the possible etiologies proposed for SR of HCC, ischemia is the most common, and systemic immunological reactions are the second cause [19,23]. A list of causes could be as follows: impairment of the hepatic circulation as a result of gastrointestinal bleeding, portal vein tumor thrombosis, blockage or reduced blood flow through arteries to the tumor as a result of a thick fibrous tumor capsule, ischemia caused by abrupt enlargement of the tumor, immune response, cessation of heavy drinking or use of herbal medicine [24]. If SR had occurred, the possibility of recurrence due to remnant viable HCC cells would have existed [25].

Concerning HCC with SR involving the peripheral blood vessels, many reports have provided evidence of arterial thrombosis, with a few noting evidence of portal vein thrombosis [18]. In the literature we have identified only a few cases of HCC in which SR was related to portal thrombosis [18,24,26,27], and just one in a patient with mixed HCC and CCA histology without portal thrombosis [28].

Similarly with HCC, we can consider that i-CCA is also susceptible to SR based on a sudden fall in hepatic blood flow due to rapid growth, arterioportal shunting, and portal vein thrombosis [29]. In our case, portal vein ligation was able to increase the arterial blood flow and help to form arterioportal shunts. The shunts could also steal the blood supply from the tumor, which might also be one of the possible causes of the observed SR.

## 4 Conclusion

Spontaneous TR is an interesting phenomenon, but its mechanisms remain unclear.

A rare case of i-CCA which showed regression after portal vein ligation was presented. Serial MRI examinations clearly demonstrated its course and morphological details.

I-CCA should be included in the tumors that could extremely rarely spontaneously regress.

## Informed consent

Written informed consent was obtained from the patient for publication of this case report and accompanying images. A copy of the written consent is available for review by the Editor-in-Chief of this journal on request.

## Ethical statement

Case reports do not require ethical approval by our institution. This study was in normal procedure with surgery consent form.

## Sources of funding

Project financed by Lucian Blaga University of Sibiu (Knowledge Transfer Center) & Hasso Plattner Foundation research grants LBUS-HPI-ERG-2023-05.

## Research registration

Name of the registry: None.

Unique identifying number or registration ID: None.

Hyperlink to your specific registration: None.

## Guarantor

D.F.C. Moga, MD, PhD.

A.A. Dan, MD.

## CRediT authorship contribution statement

**D.F.C. Moga:** Conceptualization the plan for surgery, performing the surgery, writing the original draft, reviewing the manuscript.

**G.A. Gavrilă:** Writing the literature review for case report, reviewing the manuscript.

**A.A. Dan:** Analyzing the MRI images, reviewing the manuscript.

**C.G. Smarandache:** Writing the literature review for case report, reviewing the manuscript.

## Declaration of competing interest

The authors do not have any conflicts of interest.

## Data Availability

Not applicable.
